# Unique chemical parameters and microbial activity lead to increased archaeological preservation at the Roman frontier site of Vindolanda, UK

**DOI:** 10.1038/s41598-021-94853-7

**Published:** 2021-08-04

**Authors:** C. H. Orr, R. Williams, H. H. Halldórsdóttir, A. Birley, E. Greene, A. Nelson, T. K. Ralebitso-Senior, G. Taylor

**Affiliations:** 1 School of Health and Life Sciences, Teesside University, Middlesbrough, TS1 3BX Tees Valley UK; 2 Vindolanda, Bardon Mill, Hexham, NE47 7JN Northumberland UK; 3 Faculty of Arts and Humanities, Classics Department, University of Western Ontario, 1151 Richmond St, London, ON N6A 5B8 Canada; 4 Faculty of Health and Life Sciences, Northumbria University Newcastle, Newcastle upon Tyne, UK; 5 School of Pharmacy and Biomolecular Sciences, Liverpool John Moores University, Liverpool, LS3 3AF UK

**Keywords:** Microbiology, Molecular biology

## Abstract

Waterlogged burial conditions impact upon artefact preservation. One major determinant of preservation is presence and behaviour of microorganisms, however, unravelling the mechanisms, especially in waterlogged conditions is challenging. In this study, we analysed elemental composition, bacterial diversity and community structure from excavation trenches at the Roman Site of Vindolanda, Northumberland, UK, using pXRF and 16S rRNA gene amplicon sequencing. Excavation trenches provide information of different occupation periods. The results indicated that microbial communities were dominated by *Firmicutes, Bacteroidetes* and *Proteobacteria* at a phylum level. Samples which also had visible vivianite presence showed that there were marked increases in *Methylophilus*. *Methylophilus* might be associated with favourable preservation in these anaerobic conditions. More research is needed to clearly link the presence of *Methylophilus* with vivianite production. The study emphasises the need for further integration of chemical and microbiome approaches, especially in good preservation areas, to explore microbial and chemical degradation mechanisms.

## Introduction

Vindolanda, a Roman auxiliary fort situated south of Hadrian’s Wall near Bardon Mill, Northumberland UK, is a site known for its excellent preservation of leather and wooden artefacts^1,2^. Exquisite wooden artefacts include the Vindolanda writing tablets showing the correspondence of Iulius Verecundus^3^, Prefect of the First Cohort of Tungrians, dated to between first and second centuries. The extensive assemblage of leather artefacts, especially shoes is one of the largest discovered on any Roman site, but Vindolanda has also provided the discovery of the oldest Roman boxing gloves c.a. 120 AD. The fort is situated upon the Alston formation, which is calcium carbonate sedimentary bedrock, overlaid by loamy and clayey soils. However, this alone does not explain the exceptional level of artefact preservation^2^. Between Roman occupation periods, wooden and stone buildings were destroyed, sealed with thick layers of clays and then re-built upon, forming layers in which oxygen was excluded from the decomposing material underneath. This waterlogging above dense clay layers led to the formation of anaerobic layers which are ideal preservation environments^4^.

Waterlogged environments have unique and complex hydrological chemistry^5^. Extensive monitoring at sites is a recommended and important aspect of site management to ensure artefact preservation^6^. Within waterlogged environments impacts upon artefact preservation can include, but not limited to, and be influenced by (i) redox reactions, such as Fe(III) reducing to Fe(II), and formations of chemical complexes like vivianite through combination of Fe(III) and phosphate^4^, (ii) microbiological species, such as decreasing abundance of *Proteobacteria, Actinobacteria* and *Firmicutes*, (iii) hydrological indicators, such as acidity (pH values), conductivity and oxidation–reduction potential, (iv) the decomposition material of surrounding highly organic matter, including those rich in phosphates^5^.

Redox reactions are one of the most important and visually impactful processes present at Vindolanda. Chemically vivianite (Fe_3_(PO_4_)_2_·8H_2_O) can form, when there is an availability of iron and phosphorus in the soil, lack of oxygen, low sulphur content and low pH values^4,7,8^. The phosphorus released from the decaying organic matter, such as floor material, animal faeces and plants is retained, through sorption to iron oxides and as such vivianite acts as a phosphorus sink. Upon excavation and exposure to oxygen, Fe(II) is converted to Fe(III), which produces the unique blue colouration of vivianite^4^. Interestingly, vivianite is associated with other instances of good preservation including that of human remains: for example human DNA of increased quality can be extracted from samples located within the Brisbane burial grounds from underneath vivianite crusts^7^. The formation of vivianite is also thought to be microbially mediated with studies showing a range of microorganisms capable of producing the substance in anaerobic culture when exposed to the correct conditions^8–10^. Thus, despite vivianite acting as a visible preservation indicator, the exact chemical and microbiological formation, production and generation mechanisms are not completely understood.

Microorganisms play a key role in decomposition, with above ground artefacts degraded by a range of bacteria, fungi and insects relatively quickly^11^. The speed of this degradation is impacted by oxygen availability, sample age and material which in turn impact upon the microbial community capable of carrying out decay^12^. Upon burial, particularly under anaerobic conditions, many fungal species (for example, soft rot fungi) typically involved in degradation cannot act. This means that the majority of degradation in anaerobic conditions is carried out by bacteria^13^. There is also evidence in marine sediments that key microbial communities, linked to anoxic environments can be central to increased preservation^14^. Ultimately, the mechanisms responsible for the excellent preservation observed at Vindolanda are unknown and will be explored within this study. Our assumption is that the exceptional levels of preservation seen at Vindolanda are brought about by a combination of environmental factors specifically linked to the unusual anaerobic and elemental conditions observed, through an interaction between local geochemistry, hydrology and past human activity at the site (clay sealing). Such conditions in turn impact on the microbial community within the soil, producing vivianite and limiting growth of degrading organisms.

The initial aim of this study was to characterise the microbial community within different occupation layers of archaeological soil at Vindolanda. Bacterial communities within the different occupation layers which exhibit various degrees of preservation were compared with non-occupation environmental soil layers. The overall aim was then to correlate changes in environmental variables within the site and begin to unpick whether the microorganisms, environmental conditions or a combination of the two may be responsible for the good preservation observed at Vindolanda. As the factors influencing preservation and decomposition are poorly understood it is hoped that this study will help to shape current thinking of factors responsible for improved preservation at archaeological sites, to allow strengthening of in-situ preservation techniques. To our knowledge, this is the first reported study of a Roman burial environment using both chemistry and microbiological data, obtained using next generation sequencing techniques.

## Results

### Environmental variables

pH values were consistent between the soil locations (depth1p (d1p) = 6.33 ± 0.06, depth2p (d2p) = 6.35 ± 0.20, depth3i (d3i) = 5.56 ± 0.88, depth4t (d4t) = 6.40 ± 0.16, depth5c (c1) = 6.07 ± 0.29, depth6c (c2) = 6.58 ± 0.47). Kruskal–Wallis tests showed no significant difference in pH value (χ^2^ = 46.38, df = 45, p = 0.42; Supplementary Data Figure 1) between soil locations and significant differences in moisture content (χ^2^ = 53, df = 17, p < 0.001; Supplementary Data Figure 1). Nemenyi tests showed that d1p and d2p had significantly more moisture than c1 and c2, but all other comparisons were not significantly different.

**Figure 1:**
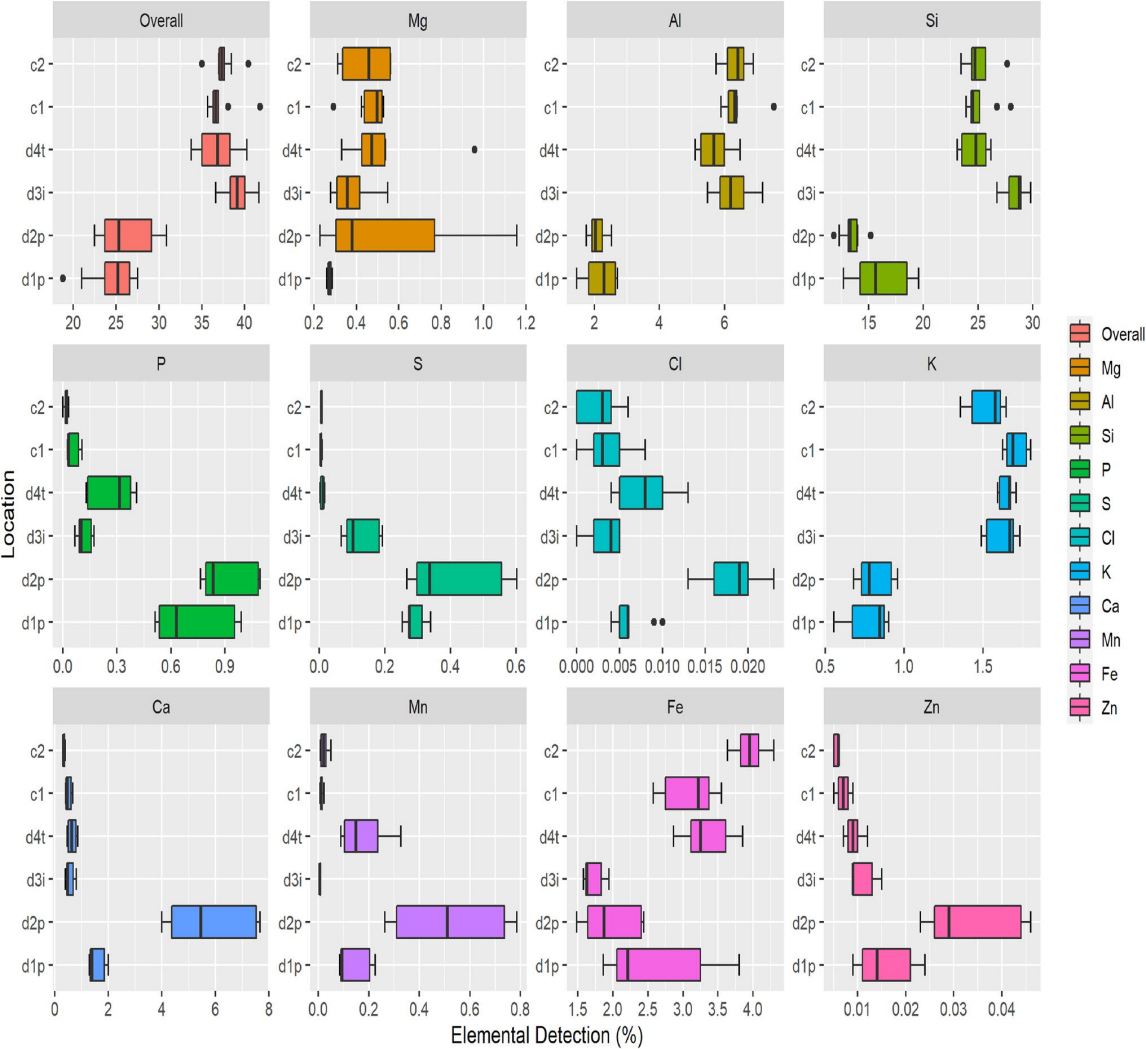
Elemental analysis for minor and major elements from sample d1p, d2p, d3i, d4t, c1 and c2.

Elemental analysis obtained from pXRF analysis showed significant changes in elemental composition between the differing locations. D1p and d2p, the sites of best preservation, displayed different elemental composition with respect to phosphorus, sulphur and iron (P < 0.001). Pairwise comparisons using Dwass–Steele–Critchlow–Fligner (DSCF) showed that the interaction between phosphorus, sulphur and iron in d1p and d2p was not significantly different to each other, but were significantly different to d3i, d4t, c1 and c2. Elemental composition for major and minor elements are provided in Fig. 1. Phosphorus has a range of Max = 1.094; Min = 0.512; Range = 0.582; Mean = 0.813; Median = 0.806 in d1p and d2p.

### Microbial community profile

**Figure 2:**
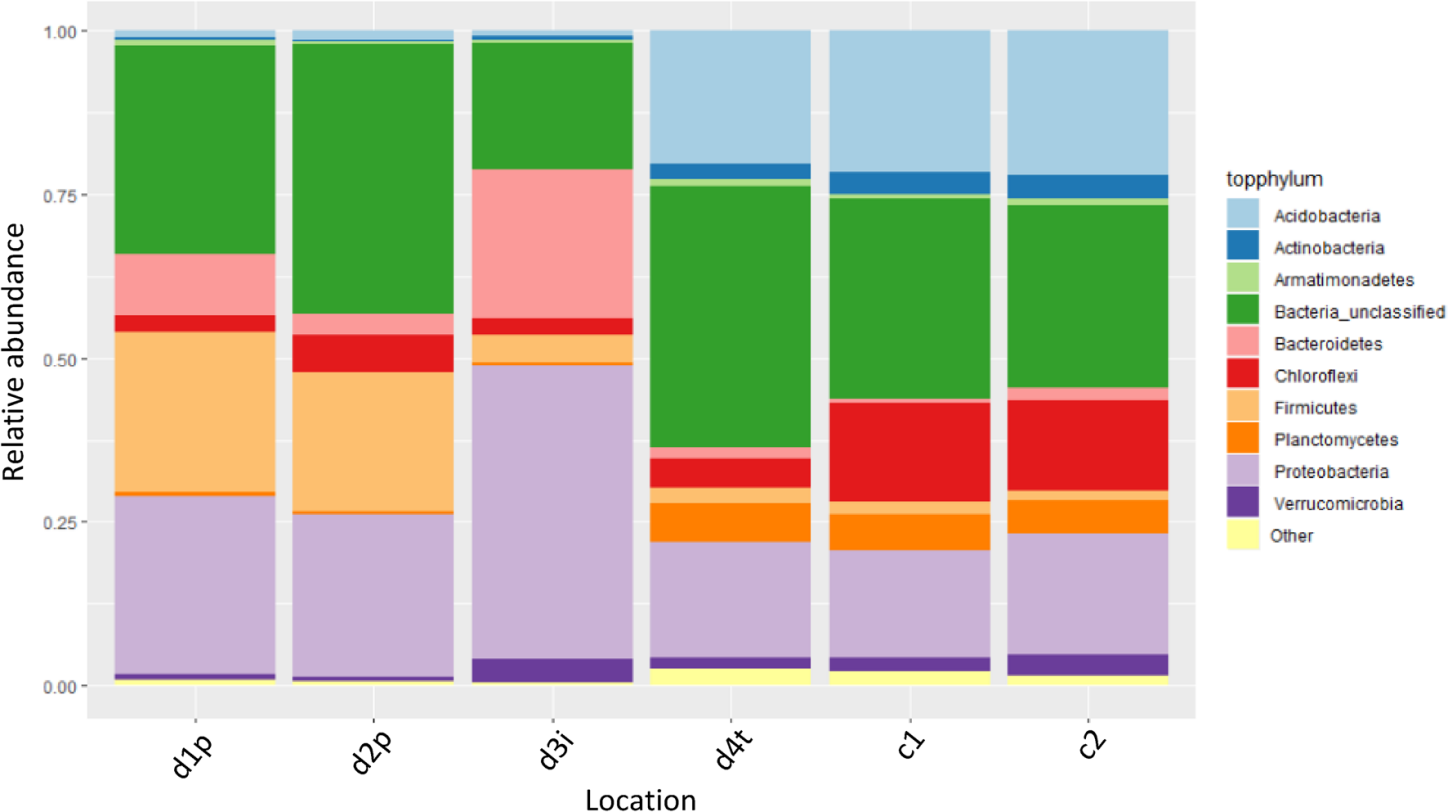
Relative abundance of bacterial phyla across six locations of the study. Replicates are averaged. The ten most abundant phyla are shown with the remainder shown as ‘other’.

There is a shift in the microbial community profile between d1p and d2p and interface layer d3i when compared with other locations (Fig. 2). Spearman’s rank correlation analysis showed significant positive correlations with depth and *Acidobacteria* (ρ = 0.825, P = 2.52 × 10^–5^), *Actinobacteria* (ρ = 0.887, P = 9,189 × 10^–7^), *Chloroflexi* (ρ = 0.724, P = 6.78 × 10^–4^) and *Planctomyces* (ρ = 0.699, P = 1.24 × 10^–3^) and negative correlations with *Firmicutes* (ρ = − 0.937, P = 9.76 × 10^–9^), *Bacteroidetes* (ρ = − 0.687, P = 1.65 × 10^–3^) and *Spirochaetes* (ρ = − 0.901, P = 3.347 × 10^–7^). There was no significant correlation linking pH values to relative abundance of any phyla.

**Figure 3:**
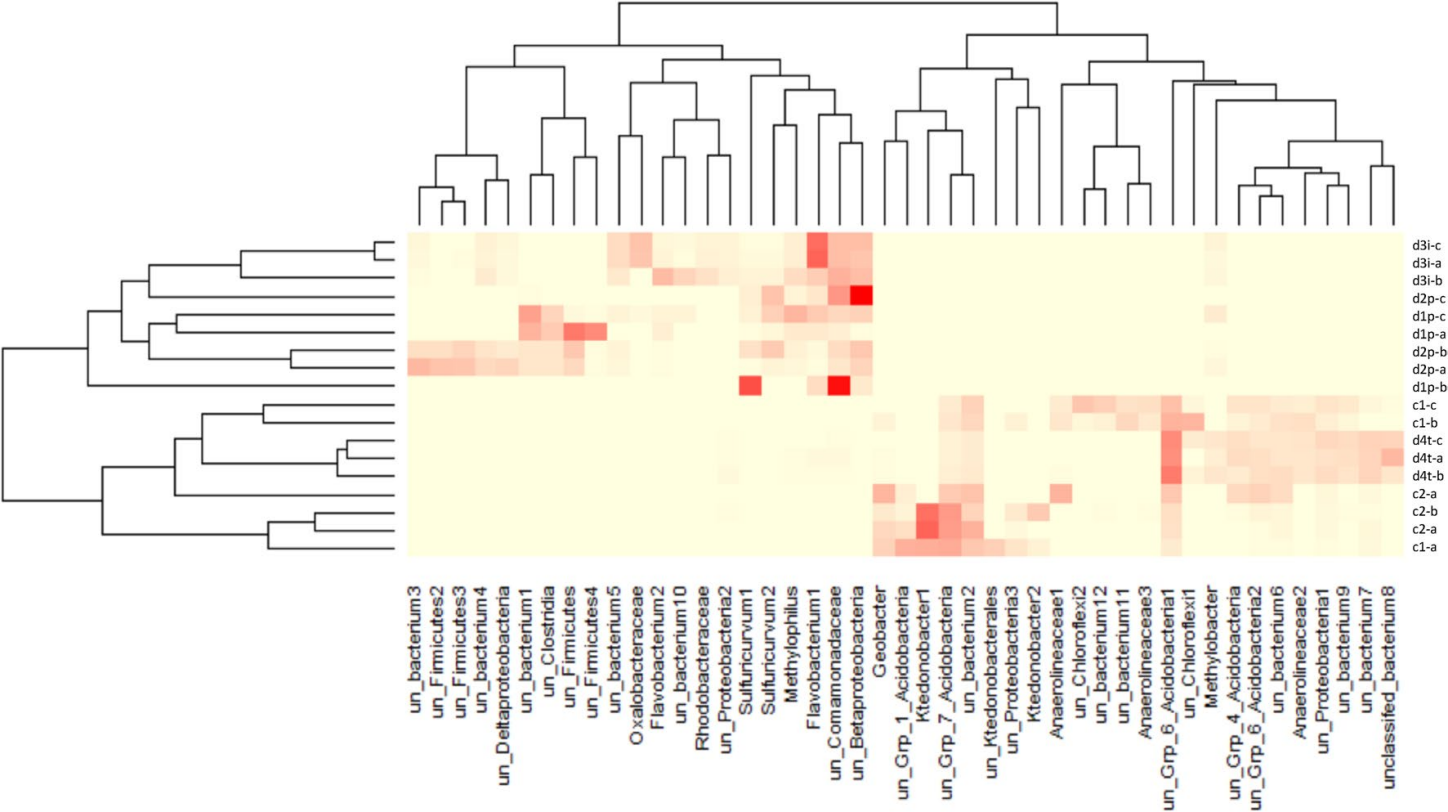
Heatmap showing the distribution of OTU within each replicate sample. Replicate samples are shown with -a, -b and -c. Data is simplified so that OTU are only depicted if they contribute more than 1% of a single sample. Dark red = 1, pale yellow = 0. Within OTU names ‘un’ = unclassified.

At OTU and genera level there were marked differences between the soil locations clearly separating samples from occupation layers with good preservation and interface samples from others (Fig. 3). Specifically, identifiable dominating genera within preservation and interface layers were *Sulfuricurvum*, *Flavobacterium* and *Methylophilus*, with *Ktedonobacter*, *Geobacter* and *Methylobacter* dominating in non-preservation layers. Although the composition of the community changes this was not reflected in a shift in diversity with both Shannon diversity and Evenness measures showing little difference between the locations: d1p H′ 5.724 ± 0.14, Evenness 0.747 ± 0.001; d2p H′ 6.340 ± 0.12, Evenness 0.773 ± 0.006; d3i H′ 5.286 ± 0.10, Evenness 0.688 ± 0.011, d4t H′ 6.741 ± 0.03, Evenness 0.825 ± 0.000; c1 H′ 5.936 ± 0.09, Evenness 0.7778 ± 0.002; c2 H′ 6.120 ± 0.09, Evenness 0.779 ± 0.009.

### Microorganisms indicative of preservation layers

**Figure 4:**
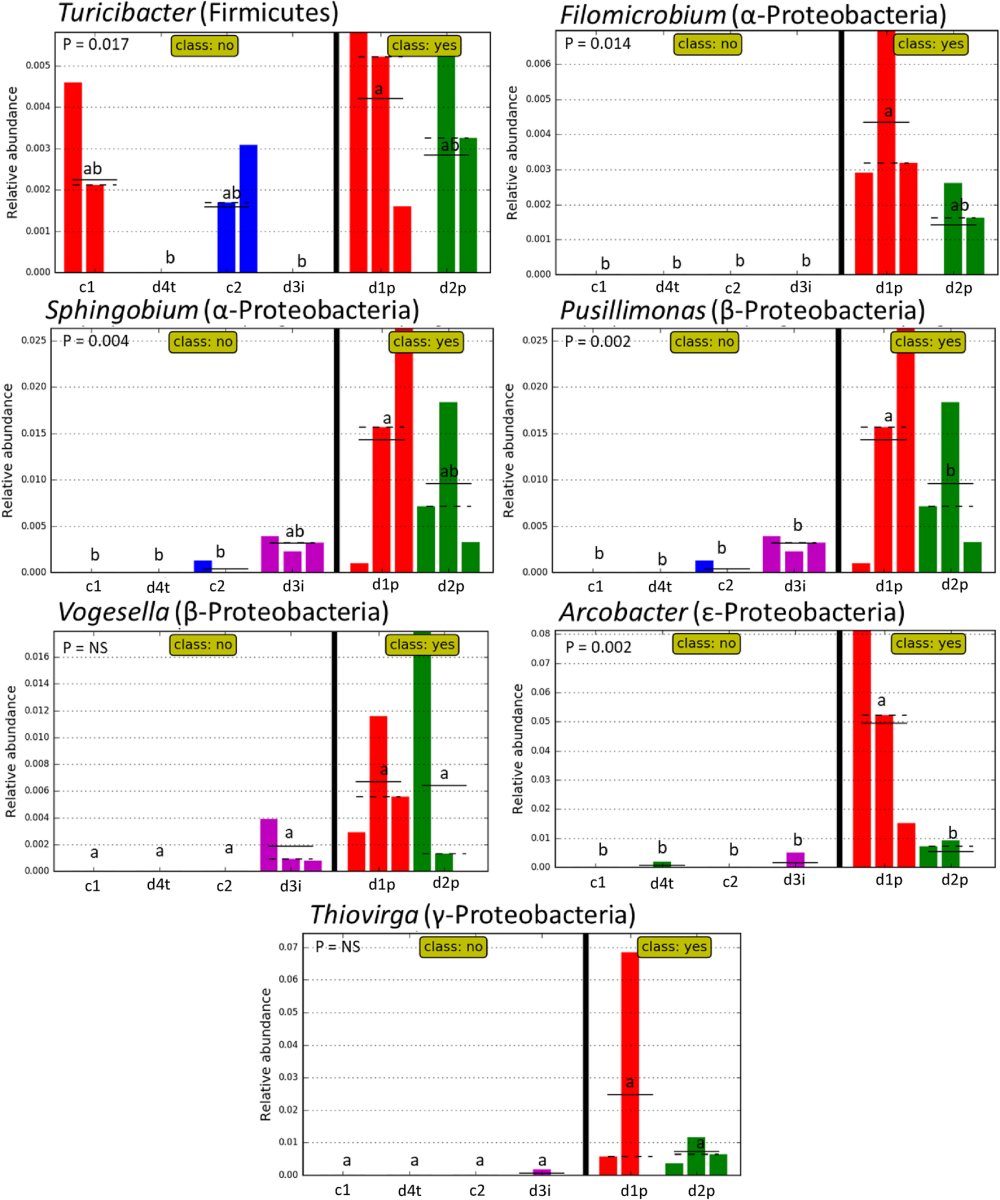
Histograms generated following LEfSe analysis showing relative abundance of genera identified as potentially indicative of preservation. Class shows if samples are within area of observed good preservation. P values are listed in corner of each figure. *NS* non-significant. Tukey’s test within each graph is represented by letters. Layers which share letters are not significantly different from each other^15^.

LEfSe analysis of the microbial community revealed specific community structure associated with preservation layers characterised by *Firmicutes, Epsilonproteobacteria, BRC1* and *Chloroflexi* with no phyla indicative of non-preservation layers due to varied communities. Tentative organisms which may be indicative of preservation layers are identified as: the Firmicute *Turicibacter*; *Alphaproteobacteria Filomicrobium* and *Sphingobium*; Betaproteobacteria *Pusillimonas* and *Vogesella*; Epsilonproteobacteria *Arcobacter;* and Gammaproteobacteria *Thiovirga* (Fig. 4).

### Composition of samples that contain vivianite

**Figure 5:**
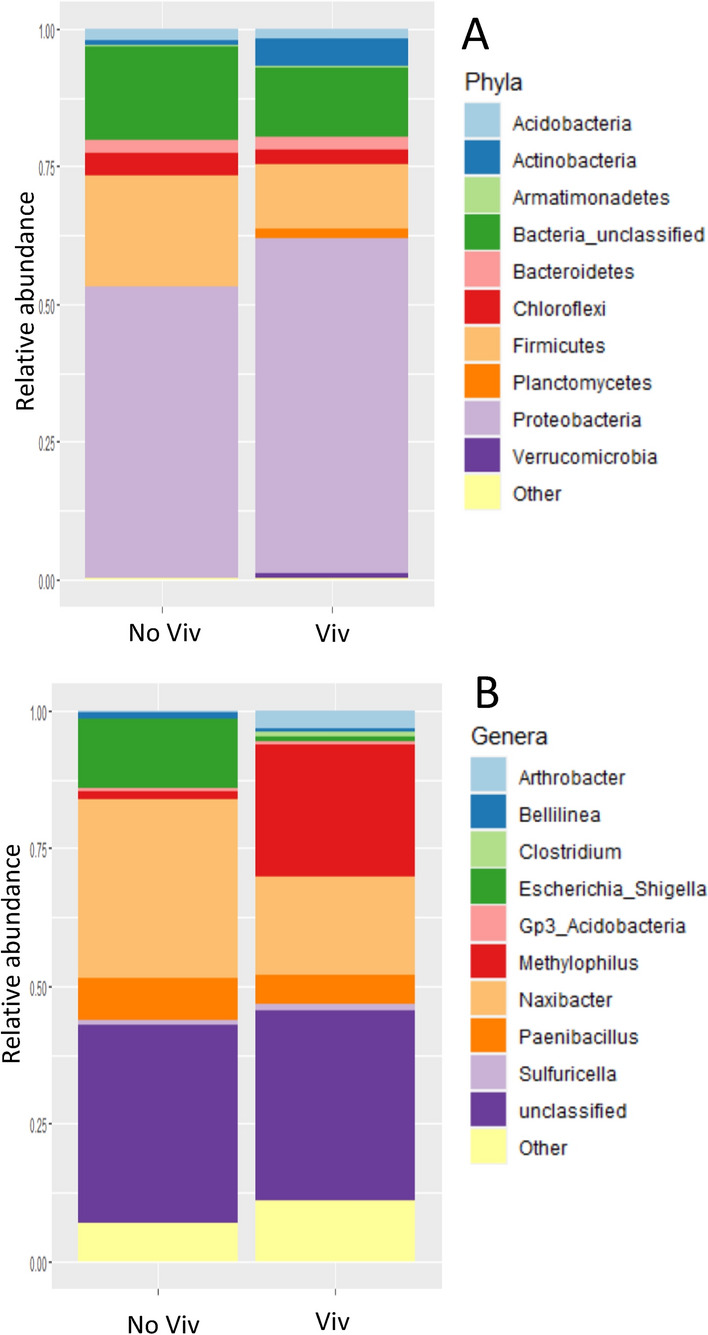
Relative abundance of bacterial phyla (**A**) and genera (**B**) within samples showing visible vivianite (Viv) or no visible vivianite (No Viv). Replicates are averaged. The ten most abundant phyla/genera are shown with the remainder shown as ‘other’.

When compared with soils within 10 cm, soils with vivianite formation showed comparable Shannon diversity (visible vivianite = 3.211 ± 1.65; no visible vivianite = 3.636 ± 1.97) and evenness (visible vivianite = 0.598 ± 0.21; no visible vivianite = 0.618 ± 0.23) at phyla level. Adjacent soils profiles were not dissimilar to the preservation layers shown in Fig. 2 (Fig. 5). However, sections with vivianite were dominated by a single genus (50.3% of the total OTU within one of the samples) *Methylophilus* compared with sections without (P = 0.0162) (Fig. 5).

## Discussion

### Elemental composition within the soil layers

Overall, there were significant shifts in elemental composition between soil samples at Vindolanda specifically in phosphorus, sulphur and iron within d1p and d2p locations. Sampling locations d3i, d4t, c1 and c2 showed consistent elemental composition regardless of the different trench locations. Whilst preservation of artefacts is poor in d3i and d4t there is evidence of Roman occupation. However, there is no such evidence in c1 and c2 suggesting they sit outside of the settlement. Interestingly, there was little observable difference within pH values between any of the samples.

Iron and sulphate reduction, denitrification and nitrate respiration are associated with anaerobic environments^16^, with dissimilatory Fe(III) reduction being the most abundant anaerobic metabolism^17^. Anaerobic soil conditions are typically characterised by the accumulation of iron cations (Fe(II) and Fe(III)) as soil-available Fe is reduced. In aerobic conditions oxidation of Fe(II) to Fe(III) seems to correlate to a speeding up of microbial metabolism and a decomposition of organic matter both within field and pot experiments^18^. However, pXRF analysis used in the current study cannot differentiate between the different oxidative states of iron; it does however, suggest that iron present within these locations is not just a change in redox state.

Phosphorus release is associated with increased iron reduction within anaerobic soil environments with the dynamics of phosphorus release and Fe(III) reduction closely linked^19^. However, there is also a relationship between the cycling of iron and phosphorus with the concentration of sulphate found within waterlogged environments. Within such conditions sulphate reduction results in increases in phosphorus release^20^. The iron and phosphorus concentrations are higher at Vindolanda within locations showing preservation and could, therefore suggest why increased vivianite are observed here^4^. Ratios of less than 1.5 sulphur:iron are needed for vivianite formation within marine sediments^21^; all soils used within this study met these conditions. However, an excess of phosphorus is also needed which is only seen in d1p and d2p.

Certain soil types are more likely to result in good archaeological preservation than others with anaerobic soils rich in organic material being more likely to show reduced biological oxidation of wood, leather and soft tissue^22^, partly due to the microorganism which flourish in such environments^12^. Reduced forms of phosphorus, sulphur and iron are more likely to be found within anaerobic soils compared with aerobic. Furthermore, the changes in moisture conditions at Vindolanda may allow reactions with organic material within the soil, thus facilitating a natural ‘tanning’ process^22^.

### The microbial community within the Vindolanda soil layers

Preservation and interface sampling d1p, d2p and d3i are distinct from other samples in terms of composition of phyla and genera. Shifts in bacterial phyla are correlated to location of sample but not pH values or elemental composition. D1p, d2p and d3i locations are dominated by *Firmicutes, Bacteroidetes* and *Proteobacteria* at a phylum level with LEfSe analysis suggesting that *Firmicutes, Epsilonproteobacteria, BRC1* and *Chloroflexi* may be indicative of preservation. Non-preservation and control locations were typified by increased abundance of *Acidobacteria, Actinobacteria* and *Planctomycetes*. Shannon diversity remained high within preservation layers and was comparable to the surrounding soil. Maintenance of diversity and evenness within good archaeological preserving environments has been shown previously in No. 1 Wangshanqiao Chu Tomb in Jingzhou, China^23^.

Previous studies have shown that at the phylum level soils within the top approximately 20 cm are likely to be dominated by *Acidobacteria, Actinobacteria* and *Alphaproteobacteria* with *Firmicutes* and *Spirochaetes* being more abundant below 50 cm^24^. *Acidobacteria* and *Actinobacteria* are increased within d4t, c1 and c2 compared with d1p, d2p and d3i. *Actinobacteria* are widely recognised as being crucial to aerobic degradation of organic matter particularly that which is cellulose based^25,26^. *Acidobacteria* are a diverse phylum which are mostly heterotrophic in metabolism and can degrade a wide range of carbohydrate sources^27^. Members of the *Acidobacteria* group 1–4 have been linked with degradation of cellulose based material^28^ and are seen in significant abundance within layers 4–6 of this study. Interestingly they are greatly reduced within the preservation layers despite the pH values remaining consistent. This is probably due to the anaerobic conditions present within these layers as the majority of *Acidobacteria* are aerobic^27^.

*Firmicutes, Bacteroidetes* and *Proteobacteria* were significantly increased within the preservation layers. However, this is most likely a result of the anaerobic conditions found rather than these phyla not being decomposers. *Proteobacteria, Firmicutes* and *Bacteroidetes* are the most dominant phyla in solid waste decomposition within anaerobic phases^29^ with *Firmicutes* and *Bacteroidetes* proposed as the main microorganisms involved in cellulose degradation^28^. There are very few studies assessing microbiology within archaeological sites. However, those available correspond with our findings of consistent microbial diversity between layers which show preservation and those which do not^23,30^. Siles also found varied soil profiles that were common to soil environments and recorded no dominant ‘rare’ microbial taxa as clear indicators of preservation^30^.

At the genus level d1p and 2dp showed increased abundance of *Sulficurvum*, *Flavobacterium* and *Methylophilus* with additional genera *Turicibacter*, *Filomicrobium*, *Sphingobium*, *Pusillimonas*, *Vogesella*, *Arcobacter* and *Thiovirga* being identified as indicative of preservation by LEfSe. These microorganisms are not typically indicative of depth or oxygen availability as changes at phylum level were. Instead *Sulficurvum*, *Flavobacterium* and *Methylophilus* are all capable of degrading polycyclic aromatic hydrocarbons (PAHs) and are found to be encouraged by increased concentrations of iron and sulphur^31,32^. *Flavobacterium* and *Methylophilus* are aerobic, whilst *Sulfuricurvum* are facultative anaerobes which are major contributors to the sulphur cycle given their abilities to oxidise sulphur and are observed in the presence of excess methane^33–35^. This coupled with the changes in soil chemistry involved suggests an unusual soil environment within the preservation layers at Vindolanda.

### Microbial community with vivianite

Interestingly, further investigation of samples with visible vivianite showed that these soils contained marked increases in *Methylophilus* compared with adjacent soils. *Methylophilus* are methylotrophic *Betaproteobacteria* commonly occurring in activated sludge, mud, and aquatic habitats, and are capable of utilizing methanol or methylamine as a sole source of carbon and energy^36^. Members of the genus are thought to be strictly aerobic and have been extracted from various soils^35^. *Methylophilus* can be found in high abundance on fresh plant material due to methanol based plant substrate availability, but would be expected to be found in low abundance within soil environments^36^. Although, outside the scope of the current study, future work should identify the occurrence and distribution of oxic/anoxic conditions, and measure CO_2_ concentrations, either at regular intervals or randomly selected points within the site, to then determine links with the dominance of specific microbial taxa, particularly *Methylophilus*.

*Methylophilus* is capable of solubilizing inorganic phosphorus^37^ and produces siderophores and indole acetic acid^35^. Studies have shown that *Methylophilus* species are capable of reducing iron species such as ferrihydrite within conditions of increased methanol with evidence that the ferrihydrite was transformed into crystalline magnetite^37^. Furthermore, other microorganisms capable of producing magnetite can also produce vivianite in phosphate rich environments^38^.

Studies in Brazil have shown that, with the exception of environments that are high in sulphur, phosphorus and iron the production of iron species such as magnetite and haematite are relatively common while the production of vivianite is more restricted^39^. These conditions are similar to those we have identified within the preservation layers at Vindolanda. Additionally, analysis within the South China Sea showed that concentrations of iron-bound phosphorus and organic-phosphorus were influenced by sulfate-driven and metal-driven anaerobic oxidation of methane resulting in the release of phosphates and Fe^2+^ and a subsequent significantly increased formation of vivianite ^40^.

## Conclusion and future work

Our findings taken together suggest that the soil at Vindolanda contains distinctive chemical and microbial profiles associated with layers that show increased preservation of organic/inorganic artefacts. These shifts are not simply the result of changes in depth and soil moisture but suggest that the specific conditions potentially allow the microbial production of vivianite which may lead to enhanced preservation of artefacts. The role of the occupation layers as phosphorus sinks is strongly linked to the abundance of vivanite. More research is needed, therefore, to clearly link the presence of *Methylophilus* with vivianite production. Since Vindolanda is complex and archaeologically important, future work should also include other key hydrogeological indicators for preservation such as conductivity and oxidation–reduction potential. Here, in situ (before any excavation) and in-field XRD can be deployed especially on pristine undisturbed parts of this Roman site to determine different oxidative states of iron. Alternatively, atmosphere controlled chambers and on-site hermetical sealing of samples could be used immediately after excavation, as demonstrated for another biogeochemically complex albeit non-archaeological context^41^.

## Methods

### Sampling and archaeological context

**Figure 6:**
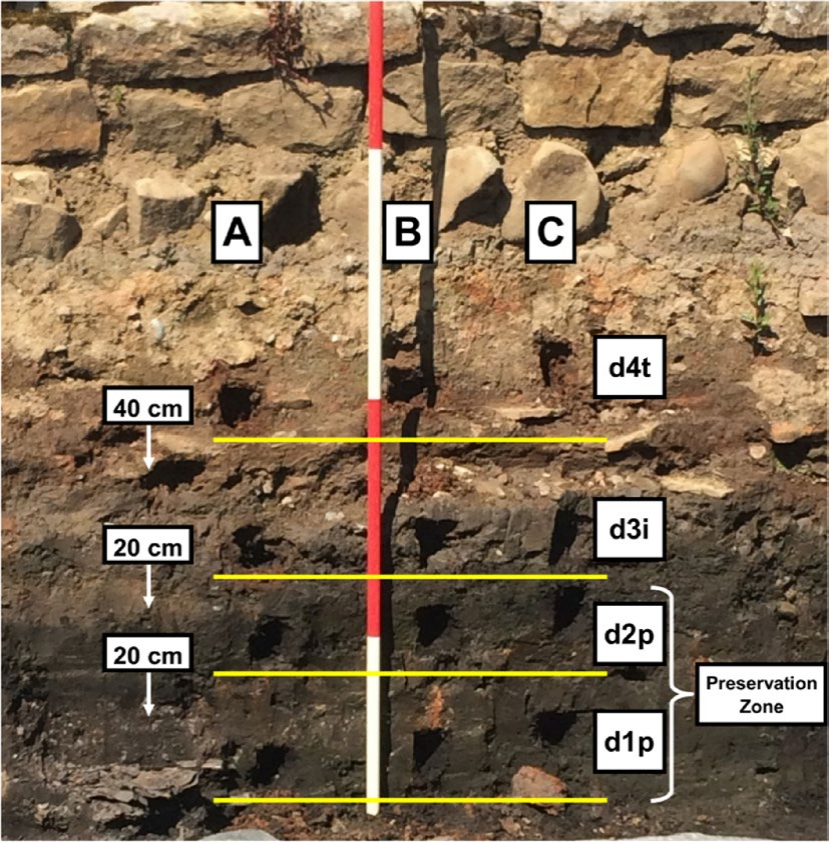
Archaeological section from the trench at Vindolanda where the samples were collected. (**A**–**C**) Replicate samples. d1–4: samples at depth (d) 1–4. Depths 1 and 2 representing the oldest archaeological contexts, associated with good preservation (p). Depth 3 is an interface (i) layer.

Soil was sampled from two trenches within the Vindolanda site (54.9911°N 2.3608°W). The external surface was scraped back by approximately 5 cm. Samples were then taken in triplicate at four equal depths (d1p, d2p, d3i and d4t), each representing a separate archaeological context (see Supplementary Figure 2). D1p and d2p correlate to the deepest layers and are associated with the best quality preservation. D3i and D4t are associated with lower quality preservation while d3i forming an interface (i) layer. The top 40 cm of the trench had was exposed for approximately 3 months prior to the sampling taking place down to the interface level of d4t. The lower 1 m section was excavated to the level of d1p four weeks prior to the sampling taking place, see Fig. 6. Soil was sampled in triplicate (A–B) from a control (c) trench showing no evidence occupation or archaeological preservation, at two depths (c1–c2) (see Supplementary Figure 2).

In addition, further samples were taken from a rampart section, as detailed in Taylor et al. The rampart sample was sampled in triplicate from the vivianite visible layer (blue) as well as triplicate samples within 10 cm which showed no visible vivianite formation.

### Moisture

Triplicate soils were weighed before and after drying in an oven overnight at 105 °C to determine the moisture content of the original sample using Eq. (1).

1 $$ wet\;weight\;(g) - dry\;weight\;(g){/}dry\;weight\;(g) \times 100 = moisture\;content\;\% $$

### pH

Soils (10 g) were placed into plastic cups with distilled water added (20 ml) for a 1:2 proportion. The solution was stirred with a glass rod and settled for 30 min. pH values was analysed using an Advanced Jenway 3510 pH meter calibrated with commercial buffers of pH 4, 7 and 10. The electrode probe was rinsed between each sample.

### pXRF

Soils were analysed following the method provided by Williams^42^. The dried soil samples were homogenised with mortar and pestle for 140 s and sieved to 2 mm. Soil were loaded into XRF sample cups (SPEX CertiPrep 3529) and covered with 5 µm polypropylene thin-film (SPEX SamplePrep 3520 window film). A Thermo Niton XL3t GOLDD + pXRF with an Ag anode (6–50 kV, 0–200 μA max X-ray tube) was used to analyse the soil. The pXRF was warmed up, system checked against the internal 1¼ Cr–½ Mo coupon, and scanned against a blank standard and NIST 2709a standard (y = 0.9674x − 0.0089, *r*^2^ = 0.9998). The NIST 2709a is a San Joaquin soil, used because it was certified for most elements of interest within the single calibration sample. The pXRF was periodically reset and system checked to account for drift. Soil samples were analysed using Mining mode (fundamental parameters), with 30-s scans for the main filter (50 kV, ≤ 50 μA), low filter (20 kV, ≤ 100 μA) and high filter (50 kV, ≤ 40 μA), and a 60-s scan for the light filter (6 kV, ≤ 200 μA). The “overall” number is the value of all detected elements combined, it is a useful value to show how much of the inorganic elemental portion of soil has been analysed across all samples. The rest of the numbers are the detections of each element individually, provided as weight%.

### DNA extraction

DNA was extracted from 0.25 g of each soil sample using the Qiagen DNeasy PowerSoil Kit following manufacturer’s instructions. DNA quality and yield were measured using NanoDrop 1000 spectrophotometer (V3.8.1, ThermoFisher) and agarose gel electrophoresis. DNA was stored at − 80 °C prior to further analysis.

### Next generation sequencing

Bacterial community DNA sequencing was made (NU-OMICs, Northumbria University, Newcastle Upon Tyne, U.K.) by targeting the V4 region of the 16S rRNA gene according to Kozich^43^. The raw sequencing reads were processed in FASTQ format and analysed with Mothur software package (version 1.36.1) (University of Michigan, U.S.A.). UCHIME was used to quality check and filter the FASTA formatted sequences for chimeras. These were aligned to the SILVA reference, and taxonomic identification of the reads was assessed by assigning sequences to operational taxonomic units (OTUs) using Ribosomal Database Project (RDP) classification. PCR negative controls were run and sequenced in parallel to the experimental samples. The OTUs recorded in negative controls and samples were excluded from further analysis. Non-bacterial sequences (Archaea/Eukaryota/Unclassified) were discarded. OTUs less than 3% were classified as rare taxa with these and the unclassified OTUs omitted from the plots.

### Statistical analysis

Data were analysed in R 4.0.2^44^ using the car, DescTools and PMCMR packages^45–47^. Normality and variance failed the Q–Q plot, Shapiro–Wilk’s and Levene’s assumption tests. Data were analysed using Kruskal–Wallis rank sum tests, followed by Nemenyi tests with Chi-Square distribution to identify significant differences between burial layers. Tukey box plots of the median and quartiles were produced using the ggplot2 package^48^ to show the change in raw elemental concentration with increasing burial depth. The tidypaleo package in tidyverse^49^ was used to produce stratigraphic plots of the moisture content and pH values against burial depth.

Operational taxonomic unit (OTU) tables and metadata were analysed using R version 4.0.2. Samples were faceted to rarefy, relative abundance data was compiled and most abundant phyla and genera were sought. Within the readr package^50^ significant differences were identified using ANOVA and TukeyHSD tests and Spearman Correlation was used to determine significant correlation between variables. Figures were created using gplots^51^. Taxa indicative of preservation were identified, and histograms generated following Linear Discriminant Analysis Effect Size (LEfSe) analysis completed in Galaxy^15^.

## Supplementary Information

Supplementary Figures.
