# Supplementary material for: Unique chemical parameters and microbial activity lead to increased archaeological preservation at the Roman frontier site of Vindolanda, UK
Source: Sci Rep. 2021 Aug 4;11:15837. doi: 10.1038/s41598-021-94853-7 (PMC8338975; doi:10.1038/s41598-021-94853-7)
Supplement: Supplementary file 1 — Supplementary Figures. [file 41598_2021_94853_MOESM1_ESM.docx]

Supplementary Figures:-

Figure 1: Stratigraphic diagram of pH level, moisture content, P, S and Fe content with sample depth. The zone of vivianite formation is highlighted in blue – between the dotted lines


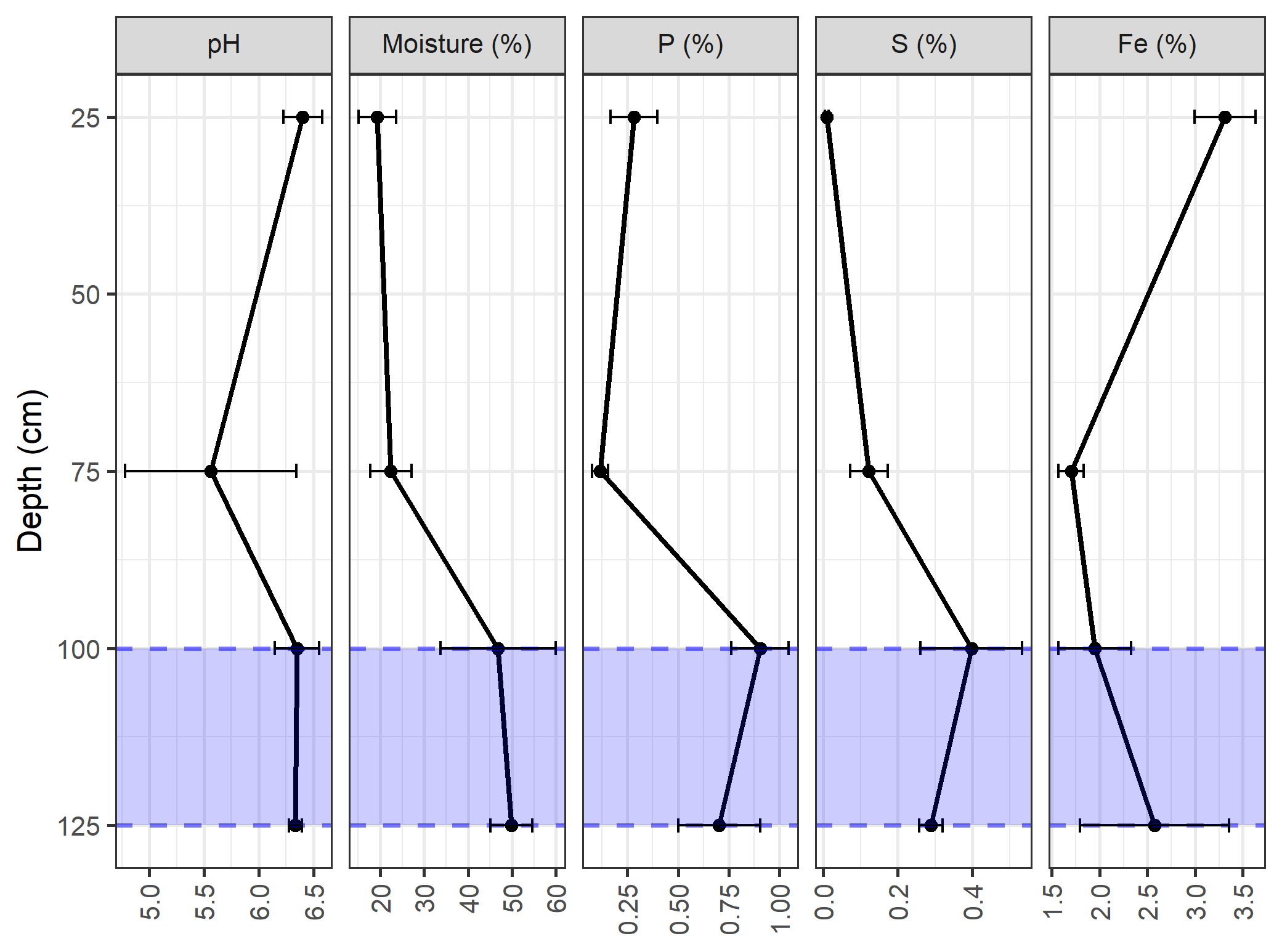


Supplementary Figure 2: Control section at Vindolanda not associated with human activity.

A-C: replicate samples.

C1-2: samples at natural control (c), taken at two depths: 1 and 2.

**
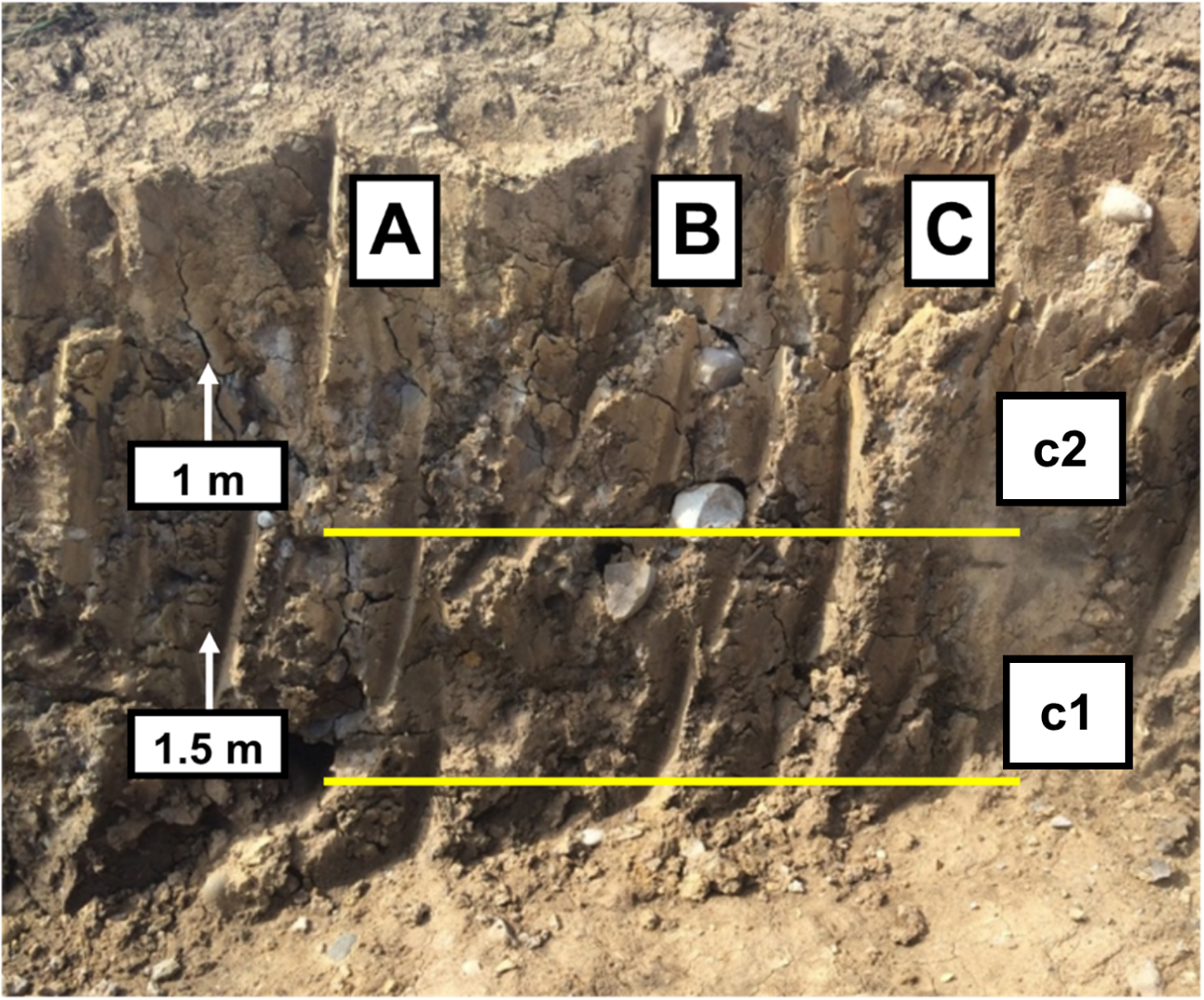
**
